# Interactions between cancer cells and bone microenvironment promote bone metastasis in prostate cancer

**DOI:** 10.1186/s40880-019-0425-1

**Published:** 2019-11-21

**Authors:** Xiangyu Zhang

**Affiliations:** Department of Pathology, Jining First People’s Hospital, Jining Medical University, No. 6 Jiankang Road, Jining, 272000 Shandong P. R. China

**Keywords:** Prostate cancer, Bone metastasis, Bone microenvironment, Colonization, Dormancy, Reactivation, Reconstruction, Nuclear factor-κB ligand, Androgen receptor, Targeted therapy

## Abstract

Bone metastasis is the leading cause of death in prostate cancer patients, for which there is currently no effective treatment. Since the bone microenvironment plays an important role in this process, attentions have been directed to the interactions between cancer cells and the bone microenvironment, including osteoclasts, osteoblasts, and bone stromal cells. Here, we explained the mechanism of interactions between prostate cancer cells and metastasis-associated cells within the bone microenvironment and further discussed the recent advances in targeted therapy of prostate cancer bone metastasis. This review also summarized the effects of bone microenvironment on prostate cancer metastasis and the related mechanisms, and provides insights for future prostate cancer metastasis studies.

## Background

Prostate cancer is one of the most common cancers in China, with an increasing incidence [1–3]. The treatment strategies of early-stage prostate cancer include surgical removal, chemotherapy, and castration therapy [4]. However, most prostate cancers will eventually develop into castration-resistant prostate cancer (CRPC). At this stage, patients usually have bone metastases, and the most common site of metastases is the vertebrae [5]. Currently, there are no effective treatments for these patients [6, 7]. To understand the limitations of the therapies that only target cancer cells, many studies have focused on the tumor microenvironment (TME), especially the interactions between cancer cells and TME [8–12]. Accordingly, in order to develop appropriate and effective therapies, gaining a comprehensive understanding of the mechanism of prostate cancer cell growth in the bone microenvironment is urgently needed.

The development of bone metastasis is a multi-step process, including (1) colonization: circulating cancer cells enter the bone marrow compartment; (2) dormancy: cancer cells adapting to the bone microenvironment and remaining dormant for a long period of time; (3) reactivation and development: cancer cells changing from the dormant state to an active proliferation state; (4) reconstruction: cancer cells changing the original bone structure and function [13, 14]. In order to provide new insights into future studies of prostate cancer metastasis, we summarized the bone microenvironment components that are involved in these four metastatic steps and their molecular mechanisms to promote bone metastasis, and subsequently discussed the recent advances in targeted therapy of prostate cancer bone metastasis.

### Functions of androgen receptor (AR) in prostate cancer bone metastasis

The normal development and function of the prostate are dependent on androgens that can activate AR signaling. Androgen and AR forms a dimer in the cytoplasm and translocates into the nucleus where AR can bind to the promoter region of target genes and regulates their transcription [15]. Androgen receptor splice variant 7 (AR-V7) is associated with drug resistance and poor prognosis in prostate cancer [16, 17]. Since androgen is important for prostate cancer development, patients with advanced prostate cancer are treated with androgen deprivation therapy (ADT), including chemical or surgical castration, which decreases the tumor size and serum prostate-specific antigen (PSA) level [18]. However, most patients will become castration-resistant and relapse after a period of time following castration [19]. During the castration-resistant phase, AR is reactivated through several mechanisms, including AR amplification and mutation, as well as activation of AR through other signaling pathways [20]. Although AR is reactivated in certain CRPC patients, AR dependence is lost in a fraction of metastases (such as neuroendocrine prostate cancer). Bone metastasis usually occurs in castration-resistant phase, thus besides ADT, the addition of AR antagonist to treatment is needed as it can effectively inhibit bone metastasis progression [21]. Androgen receptor variants (AR-Vs) that lack ligand-binding domain (including AR-V1, AR-V7, and AR-V567es) are highly expressed in bone metastases of patients with CRPC who also have high cellular-myelocytomatosis viral oncogene (c-Myc) and cyclin-dependent kinase (CDK) activity; maybe due to the crosstalk between AR-V and c-Myc [22]. It has been determined that AR plays an important role in prostate cancer bone metastasis [21, 23]. According to the activity of AR, CRPC bone metastasis can be divided into two subgroups (high and low AR activity subgroup). These two subgroups have different immune cell profiles [24].

Enzalutamide is a next-generation anti-androgen drug, which acts as an AR signaling inhibitor by competing with the natural ligands of AR (testosterone and dihydrotestosterone). It effectively treats patients with metastatic CRPC, but patients acquired drug resistance after initial sensitivity to enzalutamide. AR-V7 is one of the important reasons for the development of resistance to enzalutamide [17]. Another AR signaling inhibitor is abiraterone. It is a drug that inhibits androgen synthesis by inhibiting 17α-hydroxylase and dramatically increases the survival of patients with metastatic prostate cancer [25]. ADT is effective for prostate cancer bone metastasis patients, and its treatment effect is more obvious when combined with docetaxel [26]. Strategies to inhibit AR functions may retard bone metastasis of prostate cancer.

### Bone microenvironmental components involved in bone metastasis of prostate cancer

Bone is composed of the cortical bone and trabecular bone, in different proportions. The cortical bone forms the outer layer of bone that surrounds the bone marrow, while the trabecular bone is light and encloses the bone marrow [27, 28]. Bone is in a constant remodeling process mediated by two types of bone cells, namely the osteoclast and osteoblasts [29, 30]. The bone matrix components, such as type I collagen and mineral crystals, are very important for bone strength **[**31**]**. Most importantly, many growth factors such as insulin-like growth factors (IGFs), bone morphogenetic proteins (BMPs), transforming growth factor-beta 1 (TGFβ1), and platelet-derived growth factors (PDGFs) are found in the bone matrix [32–36]. These bone cells, bone matrix components, and growth factors have all been reported to participate in the four steps of prostate cancer metastasis. The interactions between bone cells and prostate cancer cells dominate the bone metastases formation.

#### Colonization

Bone marrow contains abundant sinusoidal vasculature, which is beneficial for circulating cancer cell migration [37]. The bone endosteum is a layer of cells lining the internal trabecular bone and is composed of hematopoietic stem cells (HSCs), osteoblasts and osteoclasts. The osteoblast niche is the site where prostate cancer metastasis first occurs [6, 38]. A previous study has shown that prostate cancer cells secrete monoamine oxidase A to influence the balance between bone resorption by osteoclasts and bone formation by osteoblasts, and ultimately promote cancer cell progression [8]. Other studies have shown that the HSC niche was the foothold of prostate cancer during their metastasis to bone, and thus plays an important role in bone metastases [39]. Another important component of bone marrow is mesenchymal stem cells (MSCs), which were found to increase the prostate cancer cell metastatic ability through suppression of AR signaling and also to affect prostate cancer cells homing to the bone marrow [40]. Therefore, colonization is the premise and basis for prostate cancer bone metastasis, which is very important.

#### Dormancy

When prostate cancer cells disseminate into the bone marrow, the bone marrow stromal cells regulate prostate cancer cells dormancy by secreting TGF-β2, MSC-derived exosomes, and thrombospondin 1 (TSP1) [41]. Exosomes are small membrane-enclosed vesicles that carry proteins, mRNAs, microRNAs, non-coding RNAs, and DNAs. Exosomes released from prostate cancer cells are taken up by bone marrow stromal cells, and then changed their gene profiling and cell signaling to promote cancer cell metastasis [42]. It has been reported that bone morphogenetic protein 7 (BMP7) promotes metastatic cancer cell dormancy by activating p38 mitogen-activated protein kinase and increasing p21 expression [43]. The Wnt/β-catenin signaling is thought to be involved in the regulation of cancer cell dormancy. Wnt5a is an important member of the Wnt/β-catenin signaling pathway, which has been reported to be involved in the inducing prostate cancer cell dormancy [44, 45]. TGF-β2 was found to induce dormancy of disseminated prostate cancer cells through upregulation of p27 and growth arrest-specific 6 (GAS6), and then re-enter the cell cycle [46]. Dormant disseminated prostate cancer cells are one important reason of cancer relapse and bone metastasis formation.

#### Reactivation and development

The dormant prostate cancer cells in the bone marrow are reactivated by sympathetic signalings, such as norepinephrine (NE), thereby the cancer cells transit into a proliferation state [47]. It has also been reported that prostate cancer cells can escape dormancy through cellular adhesion molecule [48]. Hypoxia, angiogenesis and bone resorption in the cancer-associated bone microenvironment have also been implicated in the reactivation of dormant cancer cells [49]. Since G0/G1 cell cycle arrest is one of the reasons for cellular dormancy, it was presumed that drugs or cytokines could promote cancer cell exit from the G0 phase, to enter the G2/M phase, which could induce reactivation of disseminated cancer cells and cancer development [50, 51].

#### Reconstruction

The balance between bone resorption by osteoclasts and bone formation by osteoblasts is affected by various pathological factors. When prostate cancer bone metastasis occurs, this balance is changed. The major change in bone environment is the appearance of osteogenesis, which increases new bone formation. In fact, the balance between the activity of osteoblast and osteoclast determines the phenotype and formation of metastatic lesions [52].

Osteoclast is a type of bone cell which is derived from the monocyte-macrophage hematopoietic lineage [29, 53]. Various signaling pathways play an important role in osteoclastogenesis, including pathways involving the colony-stimulating factor 1 (CSF1) and receptor activator of nuclear factor-κB ligand (RANKL) [29]. Osteoclast exerts a bone resorptive effect, involving the binding of non-osteoprotegerin (OPG)-bound, membrane-associated or soluble RANKL to RANK on osteoclasts and/or their precursors. RANKL and CSF1 are mainly secreted by bone stromal cells, including osteoblasts. They can induce bone resorption after binding to RANK on mature osteoclasts [54]. Moreover, osteoclastogenesis is regulated by the balance between RANKL and its decoy receptor, OPG. Knocking-down OPG in mice has shown to decrease the bone mineral density (BMD), whereas its overexpression was found to lead to an increase in BMD [54, 55].

Osteogenesis is the main function of osteoblasts. Osteoblasts are derived from MSCs and localize in the bone marrow stroma. The Wnt and the runt-related transcription factor 2 (RUNX2) pathways are very important regulatory pathways involved in osteoblast maturation and directional differentiation [30]. Non-collagenous proteins, such as bone gamma-carboxyglutamate protein (BGLAP; also known as osteocalcin) and secreted phosphoprotein 1 (SPP1; also known as osteopontin), and collagenous proteins, such as collagen type I, are expressed by the osteoblasts. These bone matrix proteins form the organic matrix which is then mineralized by the osteoblasts. Osteoblasts may produce some cytokines that favor the growth of prostate cancer cells. Previous studies have found that numerous osteoblasts surround the prostate cancer cells in prostate cancer bone metastases [56, 57]. Prostate cancer cells secrete BMP1, IGF, PDGF, vascular endothelial growth factor (VEGF), endothelin 1 (EDN1), plasminogen activator urokinase (PLAU) and kallikrein-related peptidase 3 (KLK3; also known as PSA), which regulate osteoblast proliferation or differentiation [56, 58]. Additionally, these factors can increase new bone matrix deposition [59, 60]. The newly formed tumor bone is woven bone with collagen fibers arranged in an irregular random structure. Moreover, the stromal cells and bone cells of the woven bone produce some factors that promote prostate cancer cell growth. Therefore, prostate cancer metastases tend to form osteoblastic lesion whereas breast cancer bone metastases are often osteolytic [56]. This is because metastatic breast cancer cells can produce local factors to stimulate osteoclasts and induce bone resorption when they invade the bone tissue [61]. Disseminated prostate cancer cells accelerate original bone damage and induce tumor woven bone formation at the end stage of metastasis.

### Mechanisms of bone metastasis in prostate cancer

There are two types of cancer bone metastases, one has a sclerotic phenotype, such as bone metastases of prostate cancer, and the other has an osteolytic phenotype, such as bone metastases of breast, lung, and renal cancers [52]. However, bone formation in prostate cancer is abnormal, as pathological fractures often occurred in prostate cancer patients.

Several factors contribute to the preference of prostate cancer cells for the bone microenvironment. The growth factors in the bone microenvironment stimulate cancer cell growth, and the cancer cells induce bone resorption and release more growth factors from the bone matrix, such as TGFβ1, which in turn induce osteoblast activity [62]. TGFβ1 could activate SMAD2 and SMAD3 in cancer cell which form complexes with transcriptional coactivators or cosuppressors to regulate gene expression [63]. This is a vicious cycle model between cancer cell and bone, but the osteoclasts remodel the bone niche to reactivate the dormant tumor cells, this is the premise of this vicious cycle [64].

Since osteoblasts express AR, and the expression of AR increases during osteoblast maturation into osteocyte, it is possible that androgen may also be involved in bone metastasis [65]. Moreover, some studies have shown that AR signaling can downregulate RANKL and inhibit osteoclastogenesis [66]. In men, low androgen levels result in bone loss and low BMD. Accordingly, patients with ADT usually have low androgen, and this may also accelerate bone metastasis [52]. Here, we summarized the molecular mechanisms of the four steps of prostate cancer bone metastasis.

#### Colonization

Previous studies have found that chemotaxis plays an important role in mediating the localization of cancer cells in bone niches. For instance, the interaction between C-X-C motif chemokine ligand 12 (CXCL12; also known as stromal-cell-derived factor 1 [SDF1]) and C-X-C motif chemokine receptor 4 (CXCR4) is the most thoroughly studied. Prostate cancer cells express CXCR4, while bone marrow stromal cells produce CXCL12, and prostate cancer cells migrate to the bone marrow partially via interaction with CXCR4 [67, 68]. The interaction of prostate cancer cell and bone marrow stromal cells has become a potential therapeutic target. Therefore, treat prostate cancer cells with AMD3100, a CXCR4 antagonist, may effectively inhibit prostate cancer cell metastasis to bone [69]. Moreover, block the interaction may be beneficial for chemotherapy, some study showed that inhibition of the interaction between cancer cells and bone stromal cells was found to increase the sensitivity of cancer cells to chemotherapy [70]. Tumor cells express integrin α_v_β_3_ and integrin α_v_β_5,_ which can specifically interact with osteopontin and integrin-binding sialoprotein (IBSP), respectively, leading to cancer cell colonization in the bone marrow [13, 71]. In addition, the interaction between cadherin 1 (CDH1) and cadherin 2 (CDH2) may also have some beneficial effect for cancer cell colonization [72, 73]. Various molecular interactions between prostate cancer cell and bone stromal cells may become the therapeutic targets for bone metastasis in future.

#### Dormancy

Dormant cancer cell is one important reason of cancer recurrence, despite the organ confined cancer was seemingly cured. Prostate cancer cells express annexin A2 receptor (ANXA2R), which binds to annexin A2 (ANX2A) that is expressed by bone cells. Additionally, GAS6/AXL axis also plays important roles in inducing cancer cells being dormant in the bone microenvironment, as prostate cancer cells express growth arrest specific-6 (GAS6) and bone express its receptor AXL (from the Greek word anexelekto or uncontrolled), their interaction leads to tumor cell dormancy [74]. The dormant state of disseminated prostate cancer cell in the bone microenvironment should be paid high attention, maybe strategies aimed at eliminating the dormant cancer cell are promising.

#### Reactivation and development

Tumor cells migrate to bone niches and become dormant in the niches for a long time until they are reactivated. Reactivation of dormant cancer cells is crucial for the whole metastatic process. Removal of the inhibitory signals plays an important role in activating the dormant cancer cells. It has been reported that vascular cell adhesion molecule 1 (VCAM1) can activate the indolent micrometastasis via the recruitment of osteoclast progenitors [75]. Drugs that inhibit osteoclast-mediated resorption can reduce tumor burden in bone metastasis, suggesting the key roles of osteoclast rather osteoblast in reactivating dormant tumor cells [14, 76].

#### Reconstruction

The RANKL/RANK/OPG pathway plays an important role in inducing the proliferation, differentiation, activation, and apoptosis of osteoclasts [54]. Human RANKL is a transmembrane protein containing a small N-terminal intracellular domain and C-terminal extracellular domain which is the characteristic feature of the TNF family. There are two forms of RANKL, one is membrane-bound, and the other form is soluble. Cleavage of the extracellular stalk region of membrane-bound RANKL by matrix metalloproteinase 14 (MMP14) and a-disintegrin-and-metalloprotease 10 (ADAM10) is the primary method for generating soluble RANKL. Alternative splicing of the RANKL transcript also contributes to the generation of soluble RANKL [77, 78]. Both forms of RANKL are bioactive and can activate osteoclasts via binding to their receptor RANK [79]. The binding of RANKL and RANK triggers the recruitment of tumor necrosis factor receptor-associated factor (TRAF) and activates the downstream signaling molecules in osteoclasts. The knockout of *TRAF6* in mice leads to severe functional impairment in osteoclasts, and causes osteoporosis [80]. The stromal cells in the bone microenvironment can secrete interleukin 6 (IL6) and RANKL, both of which can interact with osteoclasts and induce their activation and maturation [81]. RANKL signal can also activate the transcriptional factors of activator protein 1 (AP1) and nuclear factor κB (NF-κB) in osteoclasts [82]. Activation of RANK via RANKL enhances the activity of AKT serine/threonine kinase 1 (AKT1; also known as protein kinase B [PKB]) and mitogen-activated protein kinase 3 and 1 (MAPK3/MAPK1; also known as ERK1/ERK2) [83]. However, OPG can bind to soluble RANKL and counteract these effects in osteoclasts [84]. Collectively, the RANKL/RANK/OPG pathway contributes to prostate cancer bone metastasis through the activation of osteoclasts.

Endothelins (EDNs) are 21-amino acid peptides produced by endothelial and vascular muscle cells. EDN1, one of the three endothelin isoforms, is activated after proteolytic cleavage of its inactive precursor. Activated EDN1 binds to cells via its two receptors, endothelin A receptor (EDNAR) and endothelin B receptor (EDNBR), and triggers intracellular signaling. As for prostate cancer bone metastasis, EDN1 secreted by cancer cells can bind to EDNAR expressed on osteoblasts, leading to osteoblast proliferation and thereby an increase in bone density [85]. Accordingly, EDN1 seems to be a potential therapeutic target of metastatic prostate cancer, and blocking the EDN1-EDNAR signaling pathway could potentially inhibit the progression of bone metastasis. Some clinical trials have demonstrated that EDNAR inhibitors have shown success in treating bone metastasis [86, 87].

During bone metastasis, the bone resorption process needs a key enzyme to degrade the bone matrix. Cathepsin K, a lysosomal cysteine proteinase, plays an important role in this resorption process. Osteoclasts and osteoclast-like cells express abundant of cathepsin K which is involved in bone remodeling and resorption. In addition, it is well established that cathepsin K is responsible for collagen I degradation in prostate cancer bone metastasis which is necessary for tumor expansion within the bone [88, 89]. Given the important role of cathepsin K in bone resorption, it has become an attractive target in treating bone metastasis [90]. Inhibitor of cathepsin K may effectively inhibit prostate cancer metastasis and osteoclast-mediated bone resorption [91, 92]. The late stage of metastatic prostate cancer is featured with bone reconstruction, and bone pain and pathological fracture often occur, palliative treatment often implemented. Maybe treatment methods to damage the bone reconstruction process will be beneficial to patients.

### Strategies used to target bone microenvironment for therapy of prostate cancer bone metastasis

#### Bisphosphonates

Both ADT and bone metastases can lead to bone pain, spinal cord compression, and pathological fractures [93, 94]. In order to inhibit cancer cell outgrowth in the metastatic site, some strategies have been developed to target the bone microenvironment such as bisphosphonates that can bind to bone surfaces and induce osteoclasts apoptosis during bone resorption [95]. Currently, alendronate (Fosamax) [95], zoledronate (Zometa) [96], and clodronate (Bonefos) [97] are used in the clinic. Additionally, drugs such as zoledronic acid were found to effectively reduce bone pain and pathological fracture in prostate cancer patients with bone metastasis and could increase the overall quality of life of these patients [98]. However, despite patients being treated with bisphosphonate, a large proportion of patients still have skeletal-related events (SREs) and these agents are associated with several safety and tolerability concerns [95]. Nephrotoxicity is a side effect of bisphosphonates. Thus, renal monitoring is necessary in these patients treated bisphosphonates. Another potential bone-targeting drug, namely OsteoDex is a macromolecular polybisphosphonate which can effectively inhibit osteoclasts and has anti-tumor ability. This drug does not have serious adverse effects compared with alendronate [99]. Bisphosphonates is very clinically effective to alleviate the patient’s bone pain and improve their life quality.

#### Monoclonal antibody

Denosumab is an IgG2 monoclonal antibody that targeting RANKL. It can effectively inhibit osteoclastogenesis in bone metastasis and delay the onset of SREs [100]. Denosumab has a high affinity for human RANKL and a long half-life. It was developed for treating SREs induced by bone metastasis and multiple myeloma [101]. Denosumab can only bind to human RANKL, both soluble and membrane-bound primate RANKL, but fails to recognize rodent RANKL. Additionally, it cannot bind to other TNF family members, such as RANK, CD40 ligand (CD40LG), and TNF, but can bind to the DE loop region of human RANKL (this region can interact with RANK on osteoclasts) [102]. Some clinical studies have shown that denosumab was superior to zoledronic acid in treating bone metastasis [103, 104]. Denosumab was found to reduce osteoclast activity and inhibit bone turnover, therefore, the bone mass and density increased significantly in bone metastasis patients treated with denosumab [105]. Recently, studies have shown that denosumab prolonged prostate cancer patients’ bone-metastasis-free survival and delayed the onset of the initial bone metastasis [103]. Unlike zoledronic acid, denosumab has almost no side effects, thus patients do not require renal monitoring or dose adjustments [106]. However, denosumab associated severe hypocalcemia is one clinical problem that needs to be paid attention. More work should be done to decrease the side effect of denosumab.

#### Bone-targeted nanomedicines

A reported study used a copolymer of polyethylene glycol (PEG) to encapsulate the chemotherapeutic agent doxorubicin to prepare nanoparticles for skeletal metastases treatment. In this study, the nanoparticles were also modified with alendronate for targeting bone [107]. Indeed, in vitro and in vivo studies have shown that this drug delivery system has a good bone-targeting ability and treatment effect [108]. Another study using polylactic-co-glycolic acid (PLGA) nanoparticle, anchored with zoledronate as a carrier loaded with docetaxel, has shown good bone-targeting ability. The zoledronate tagged nanoparticles had enhanced bone retention, and can effectively kill cancer cells [109].

Another promising target for treating prostate cancer bone metastasis is folate hydrolase 1 (FOLH1; also known as prostate-specific membrane antigen [PMSA]). FOLH1 is expressed by prostate cancer cells. RNA aptamer A9 can specifically bind to FOLH1. Accordingly, some studies using RNA aptamer A9-modified micelles to treat bone metastasis have achieved good therapeutic results, and this drug delivery system also had well targeting effect [110]. In another study, calcium phosphate nanoparticles modified with alendronate-conjugated PEG for bone metastasis treatment was used and demonstrated that this drug delivery approach had good biocompatibility, biodegradability, and bone-targeting ability [111]. Radiopharmaceuticals are also promising agents for the treatment of bone metastasis due to their bone-seeking property which makes them suitable for treating the bone lesions [112]. Additionally, radium-223-chloride has been shown to be beneficial for the overall survival of the prostate cancer patients with bone metastasis [113]. Other radiopharmaceuticals, such as rhenium-188-HEDP and 177Lu-EDTMP, were effective in relieving the bone pain for prostate cancer patients with bone metastasis [114]. Currently, more and more novel radiopharmaceutical agents are being developed for prostate cancer bone metastasis. In general, bone targeted nanoparticles can delivery more than one type drug at one time, can specially come to the metastatic foci, which can also evade drug resistance. They are a very promising method to treat metastatic prostate cancer.

### Remaining questions

**Fig. 1:**
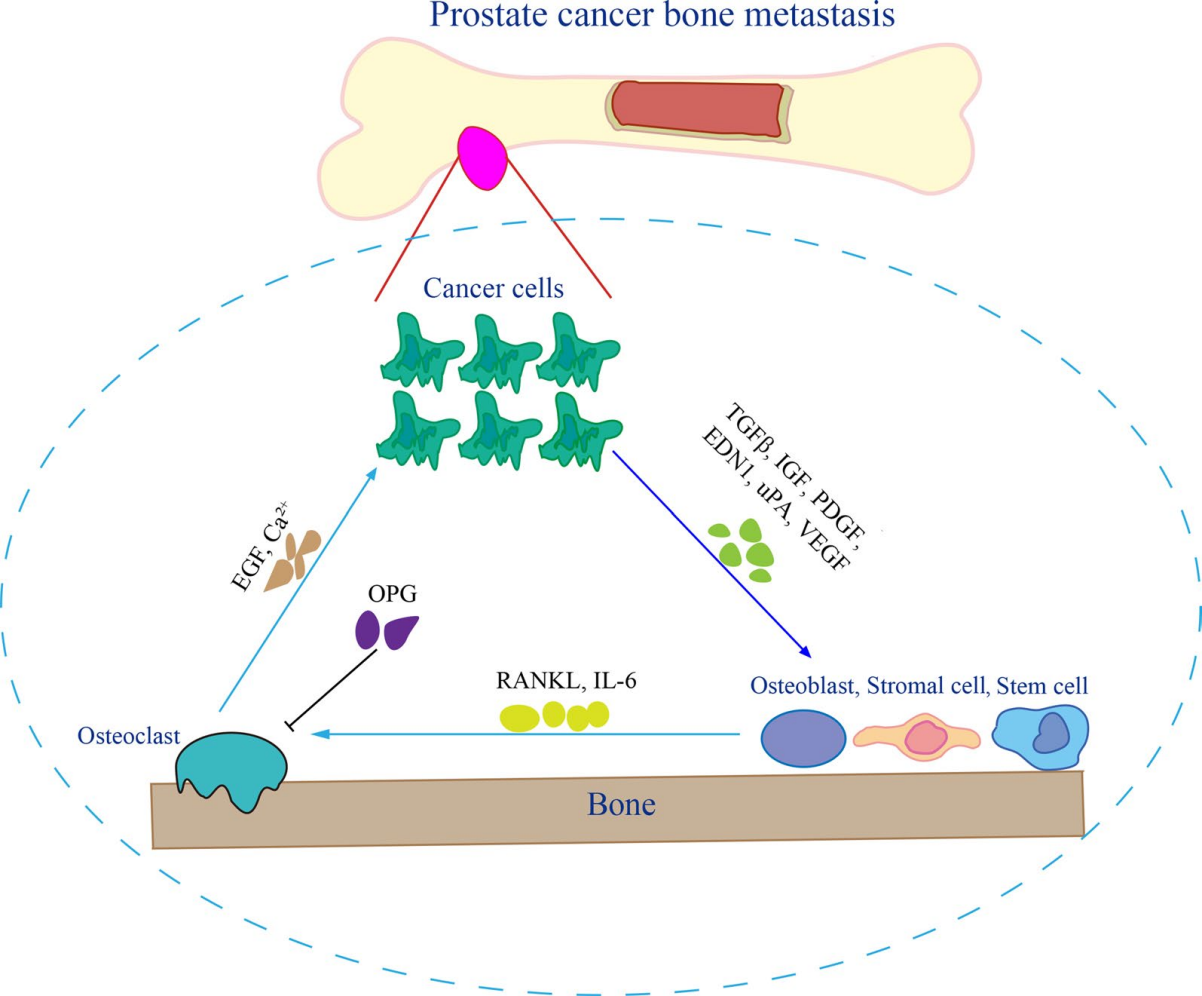
Vicious circle in the bone microenvironment of bone metastatic prostate cancer. Prostate cancer cells secrete TGFβ, IGF, PDGF, EDN1, uPA, and VEGF, which regulate osteoblast proliferation and/or differentiation. Osteoblasts secrete RANKL and IL-6 to activate osteoclasts, while OPG can inhibit the activation of osteoclasts, which elicits bone resorption and secrete EGF and calcium, thereby stimulating cancer cell proliferation in bone. Abbreviations: TGFβ, transforming growth factor β; IGF, insulin-like growth factor; PDGF, platelet-derived growth factor; EDN1, endothelin 1; uPA, urokinase plasminogen activator; VEGF, vascular endothelial growth factor; RANKL, receptor activator of the nuclear factor-κB ligand; IL-6, interleukin 6; OPG, osteoprotegerin; EGF, epidermal growth factor

Metastatic prostate cancer is the leading cause of death among prostate cancer patients and a burden on the public health system [115]. The interaction of metastatic cancer cells and bone microenvironmental cells is very complex. Therefore, current therapy does not seem to be effective enough to treat prostate cancer bone metastasis. A deeper understanding of the bone microenvironment, especially the in-depth understanding of the vicious cycle pathway involved in the bone microenvironment, insight into the bone microenvironment (Fig. 1), has provided some insights into this issue. Moreover, reactivation of dormant cancer cells in bone metastasis is the main reason of cancer recurrence but the molecular mechanisms that regulate cancer cell dormancy remain poorly characterized. The mechanism underlying cancer cell reactivation is also very important for cancer cell growth in the bone which needs to be more clearly elucidated. A better understanding of these mechanisms will lead to the design of better targeted therapy for bone metastatic prostate cancer, of which Denosumab is a successful example. However, drug resistance is also an intractable problem during the treatment of bone metastatic prostate cancer. Adaptive treatments based on mathematical modeling for prostate cancer treatment have achieved encouraging outcomes in clinical trials [116, 117]. It is possible that mathematical modeling could be used for elucidating the interaction of cancer cells and bone cells in the future.

### Future directions

Novel targeted therapies aimed at the key mechanism of prostate cancer bone metastasis should be developed, and the combination therapy using different treatment modalities is a promising strategy. Therapies that target the dormant cancer cell in the bone will be beneficial for cancer metastasis treatment.

## Conclusions

Prostate cancer is prone to lead to bone metastasis which mainly involves four steps, namely cancer cell colonization, dormancy, reactivation and development, and bone reconstruction. The interactions between cancer cells and bone cells play important roles during these complex processes. Accordingly, in this review, the bone microenvironment components, mechanisms of bone metastasis, as well as targeted therapies for prostate cancer bone metastasis were discussed. A deeper understanding of the reactivation mechanism of dormant prostate cancer cells would be very helpful for the development of bone metastasis treatments. Targeted therapies based on the bone metastasis mechanism may be developed in the future to prevent bone metastasis in cancer.

## Data Availability

Not applicable.

## Abbreviations

CRPC: castration-resistant prostate cancer
TME: tumor microenvironment
AR: androgen receptor
AR-V7: androgen receptor splice variant 7
ADT: androgen deprivation therapy
PSA: prostate-specific antigen
AR-V: androgen receptor variant
c-Myc: cellular-myelocytomatosis viral oncogene
CDK: cyclin-dependent kinase
IGF: insulin-like growth factor
BMP: bone morphogenetic protein
TGFβ1: transforming growth factor-beta 1
PDGF: platelet-derived growth factor
HSC: hematopoietic stem cell
MSC: mesenchymal stem cell
TSP1: thrombospondin 1
BMP7: bone morphogenetic protein 7
GAS6: growth arrest-specific 6
NE: norepinephrine
CSF1: colony-stimulating factor 1
RANKL: receptor activator of nuclear factor-κB ligand
OPG: osteoprotegerin
RANK: receptor activator of nuclear factor-κB
BMD: bone mineral density
RUNX2: runt-related transcription factor 2
BGLAP: bone gamma-carboxyglutamate protein
SPP1: secreted phosphoprotein 1
VEGF: vascular endothelial growth factor
EDN1: endothelin 1
PLAU: plasminogen activator urokinase
KLK3: kallikrein-related peptidase 3
CXCL12: C-X-C motif chemokine ligand 12
CXCR4: C-X-C motif chemokine receptor 4
IBSP: integrin binding sialoprotein
SDF1: stromal-cell-derived factor 1
CDH1: cadherin 1
CDH2: cadherin 2
ANXA2R: annexin A2 receptor
ANX2A: annexin A2
GAS6: growth arrest- specific 6
AXL: anexelekto
VCAM1: v ascular cell adhesion molecule 1
MMP14: matrix metalloproteinase 14
ADAM10: a-disintegrin-and-metalloprotease 10
TRAF: tumor necrosis factor receptor-associated factor
IL6: interleukin 6
AP1: activator protein 1
NF-κB: nuclear factor κB
PKB: protein ki nase B
AKT1: AKT serine/threonine kinase 1
MAPK: mitogen-activated protein kinase
EDN: endothelin
EDNAR: endothelin A receptor
EDNBR: endothelin B receptor
SRE: skeletal-relat ed event
CD40LG: CD40 ligand
PEG: polyethylene glycol
PLGA: polylactic-co-glycolic acid
FOLH1: folate hydrolase 1
PMSA: prostate-specific membrane antigen
